# Rapid Detection and Antibiotic Susceptibility of Uropathogenic *Escherichia coli* by Flow Cytometry

**DOI:** 10.3390/microorganisms8081233

**Published:** 2020-08-13

**Authors:** Alexandra Mihaela Velican, Luminiţa Măruţescu, Crina Kamerzan, Violeta Corina Cristea, Otilia Banu, Elvira Borcan, Mariana-Carmen Chifiriuc

**Affiliations:** 1 Department of Botany and Microbiology, Faculty of Biology, University of Bucharest, 60101 Bucharest, Romania; velican.alexandra@yahoo.com (A.M.V.); carmen.chifiriuc@gmail.com (M.-C.C.); 2 Research Institute of the University of Bucharest, 050095 Bucharest, Romania; crina.saviuc@yahoo.com; 3 Synevo Central Laboratory, Medicover, 021408 Bucharest, Romania; dr.violetacristea@gmail.com; 4 University of Medicine and Pharmacy “Carol Davila”, 050474 Bucharest, Romania; 5 Institute of Cardiovascular Diseases Prof. C.C. Iliescu, 022322 Bucharest, Romania; otiliabanu@gmail.com; 6 Clinical Institute Fundeni, 22328 Bucharest, Romania; elvinico@yahoo.com

**Keywords:** flow cytometry, antibiotic susceptibility, *E. coli*, urine, rapid detection

## Abstract

Background: Early preliminary data on antibiotic resistance patterns available before starting the empiric therapy of urinary tract infections (UTIs) in patients with risk factors for acquiring antibiotic resistance could improve both clinical and epidemiological outcomes. The aim of the present study was two-fold: (i) to assess the antibiotic susceptibility of uropathogenic *Escherichia coli* isolates, exhibiting different antibiotic resistance phenotypes, directly in artificially contaminated urine samples using a flow cytometry (FC) based protocol; (ii) to optimize the protocol on urine samples deliberately contaminated with bacterial suspensions prepared from uropathogenic *E. coli* strains. Results: The results of the FC based antimicrobial susceptibility testing (AST) protocol were compared with the reference AST methods results (disk diffusion and broth microdilution) for establishing the sensitivity and specificity. The proposed FC protocol allowed the detection and quantification of uropathogenic *E. coli* strains susceptibility to nitrofurantoin, trimethoprim–sulfamethoxazole, ciprofloxacin, and ceftriaxone within 4 h after the inoculation of urine specimens. The early availability of preliminary antibiotic susceptibility results provided by direct analysis of clinical specimens could essentially contribute to a more targeted emergency therapy of UTIs in the anticipation of AST results obtained by reference methodology. Conclusions: This method will increase the therapeutic success rate and help to prevent the emergence and dissemination of drug resistant pathogens.

## 1. Introduction

The increasing prevalence of resistant uropathogens highlights the acute need of rapid antibiotic susceptibility testing (AST) assays for improving the empiric broad-spectrum antibiotic therapy results. The rapid detection of antibiotic resistance, before the standard susceptibility test results are provided, could contribute on one side, to a tailored antibiotic treatment with favorable clinical effects and on the other one, to the decrease of inappropriate antibiotic usage that may further limit the emergence and spread of drug resistant pathogens. Currently, the microbiological diagnosis of urinary tract infections (UTIs) is based on isolation and quantification of the uropathogenic strains on agar plates followed by microbial identification, usually requiring 18–30 h. An additional 18–24 h are needed for providing antibiotic susceptibility results. The standard AST methods based on the assessment of microbial growth inhibition include manual (e.g., disk diffusion, broth microdilution) and automated AST, such as the Microscan Walkaway (Beckman Coulter), Phoenix Automated Microbiology System (BD), and Vitek 2 (bioMérieux) [1]. Several analysis protocols based on flow cytometry (FC) were developed for microbial detection and species identification, as well as for AST and elucidation of antimicrobial resistance mechanisms [2,3]. The major advantage of using FC directly on clinical specimens is that it could provide early data on antibiotic susceptibility profiles, thereby allowing the implementation of an adequate empirical treatment [4]. Many studies demonstrated that automated FC instruments could assure rapid screening of positive urine samples thus reducing the number of analyzed samples in clinical settings and improving diagnosis of UTI [5,6,7,8,9,10]. In our previous research, we have used FC for assessing the antimicrobial activity of several chemical and natural extracts [11,12,13]. In the present study, in order to address the accelerating levels of antibiotic resistance rates in uropathogens, we evaluated the potential of FC for the preliminary AST directly in urine specimens. This method was tested on urine samples artificially contaminated with uropathogenic *Escherichia coli* strains and the results were compared with those obtained by broth microdilution and disk diffusion methods.

## 2. Materials and Methods 

### 2.1. Bacterial Strains 

A total of 29 *E. coli* strains harboring different resistance phenotypes were recovered from urine specimens of hospitalized patients and identified by mass spectrometry using the MALDI–TOF MS Biotyper. All patients agreed to participate in the study by providing clinical samples and written informed consent. The study was approved by the Ethical Committee of the hospital unit (Ethical Committee Approval no 164/05.12.2017). The *E. coli* ATCC 25922 was used as control strain. All bacterial strains were maintained in the laboratory on nutrient agar. 

### 2.2. Antibiotics and Standard Methods for Antibiotic Susceptibility Testing

Disk diffusion method was performed using commercial antibiotic disks and Muller–Hinton agar, according to the CLSI recommendations [14]. Minimum inhibitory concentration (MIC) was determined for four antibiotics, i.e., ceftriaxone, ciprofloxacin, nitrofurantoin, and trimethoprim–sulfamethoxazole (Sigma–Aldrich), by using the 96-well broth microdilution (CLSI, 2018) method. Stock antibiotic solutions were prepared accordingly with CLSI and conserved at −20 °C. The broth microdilution testing domain was of 4 to 1 µg/mL for ciprofloxacin, 4 to 1 µg/mL for ceftriaxone, 150 to 18 µg/mL for nitrofurantoin, and 100 to 25 µg/mL for trimethoprim–sulfamethoxazole. All panels were incubated at 37 °C and read after 18–24 h. The lowest antibiotic concentration that completely inhibited the visible microbial growth and was confirmed by the spectrophotometer reading was recorded as the MIC value. 

### 2.3. FC Based Antibiotic Susceptibility (FC AST) Testing Protocol

Fresh bacterial cultures obtained on solid medium were used for the preparation of 1.5 × 10^8^ CFU (colony forming units)/mL suspensions in sterile phosphate buffer saline (PBS) that were further diluted 1:100 in a sterile urine sample (the same urine sample specimen microbiologically confirmed as sterile was used in all the experiments, in order to assure the homogeneity of the results) and 1:100 in Muller–Hinton broth, respectively. For FC AST, three different concentrations of antibiotics ((ceftriaxone (4, 2, and 1 μg/mL), ciprofloxacin (4, 2, and 1 μg/mL), nitrofurantoin (150, 75, and 18 μg/mL) and trimethoprim–sulfamethoxazole (100, 50, and 25 μg/mL)) were prepared in broth 1:1 from stock solutions and aliquoted in 96-wells plates, which were further inoculated with an equal volume of bacterial suspension, for reaching a final density of 5 × 10^5^ CFU/mL. The 29 clinical strains and *E. coli* ATCC 25922 reference strain were characterized by FC, before using them to contaminate the urine sample, in order to optimize the staining protocol. Wells containing bacteria not exposed to antibiotics and uninoculated wells, containing only broth, served as positive growth and negative controls, respectively. After 4 h of incubation at 37 °C, the bacterial cells were stained with DiBAC4(3)[(bis–(1,3– dibutylbarbituric acid) trimethineoxonol], a membrane potential sensitive dye (Invitrogen/Life technologies, Carlsbad, USA) (absorption 540 nm, emission 590 nm), at a final concentration of 0.5 μg/mL [15]. After a minimum of 30 min incubation in the dark, at room temperature, the median intensity of fluorescence (MIF) was measured with an Accuri C6 plus flow cytometer (Becton Dickinson, Biosciences) in the channel of fluorescence FITC (filters 530/30 nm). For the FC analysis of bacterial population, a total of 10,000 events in the defined bacteria gate were acquired. For each strain, non-treated and non-stained cells were also analyzed in order to evaluate the native cell auto-fluorescence and to define the acquisition settings. Data acquisition and analysis were carried out with BD Accuri C6 plus and FlowJo 10.4.2. 

### 2.4. FC AST Data Acquisition and Analysis

The effects of the tested antibiotics on *E. coli* isolates were assessed using DiBAC4(3) staining and FC measurements. Bacterial cells not exposed to antibiotics were detected based on their light scatter characteristics, after 4 h of incubation, both in urine samples and broth. The bacterial growth control served for establishing the first acquisition gate used to define the localization of bacterial cells population, based on forward and side scatter properties, and to exclude background signals and debris. The green fluorescence measurements were performed only for this gate. DiBAC4(3) dye was used for the detection of bacterial membrane potential alterations caused by antibiotic treatment. The fluorescence distribution of the stained growth control, not exposed to antibiotic, was always located in the first decades of the logarithmic scale, thus corresponding to a low fluorescence signal (polarized cells). The acquired low fluorescence events were used to define a second gate on the fluorescence histograms. The fraction of the bacterial cell population that exhibited an increased green fluorescence signal (loss of membrane polarization) was calculated based upon the exclusion of the fluorescence events corresponding to the untreated growth control, in the second gate. For each selected antibiotic concentration, a staining index (SI) was calculated as the ratio of fluorescence intensity of bacterial cells treated with antibiotic to that of non-treated cells. An increased median intensity of the green fluorescence (530/30 nm), of at least twice corresponded to dead bacterial cells (depolarized cells) [3].

### 2.5. Data Analysis

The results of the FC AST protocol were analyzed by comparison with the reference AST methods results (disk diffusion and broth microdilution). Sensitivity and specificity of the proposed FC protocol was calculated using the formulas a/(a + c) and d/(b + d), respectively, where a is the number of true resistant isolates correctly confirmed by the FC method, c is the number of true resistant strains that were assigned susceptible by the FC method, d is the number of true susceptible isolates that were correctly confirmed by the FC method, and b is the number of true susceptible isolates that were identified as antibiotic resistant by FC. Broth microdilution was considered the gold standard method. A major discrepancy was considered when the *E. coli* isolate was classified as resistant by the FC AST method and susceptible by the standard methods (false-resistant or false-positive result). For major discrepancy rate calculations, the denominators were 29 *E. coli* susceptible to nitrofurantoin, 22 *E. coli* susceptible to trimethoprim–sulfamethoxazole, 17 *E. coli* susceptible to ceftriaxone, and 13 *E. coli* susceptible to ciprofloxacin. A very major discrepancy was considered when the isolate was classified as susceptible by the FC AST method and resistant by the standard methods (false-susceptible or false-negative results). For very major discrepancy rate calculations, the denominators were seven *E. coli* resistant to trimethoprim–sulfamethoxazole, 12 *E. coli* resistant to ceftriaxone, and 16 *E. coli* resistant to ciprofloxacin. If the MIC calculated by the FC AST method was within a single two-fold dilution of the MIC provided by the standard broth dilution method, the results of the two methods were considered to be in agreement [16].

### 2.6. Analysis of Clinical Urine Samples Using the FC AST Protocol 

In order to evaluate the appropriateness of the FC AST protocol for the detection of uropathogenic bacteria and rapid testing of their antibiotic susceptibility, a total of 117 urine samples collected from inpatients (55 female and 62 males), were analyzed by FC in parallel with the standard urine culture methods. All patients agreed to participate in the study, providing urine samples and signing the informed consent. The study was approved by the Ethical of the University of Bucharest (Ethical Committee Approval no 164/05.12.2017). 

For the FC AST analysis, briefly, a volume of 50 µL from each urine sample was added, in triplicate, to 50 µL antibiotic solution, prepared in broth. Two antibiotics were tested, i.e., ceftriaxone (4 μg/mL) and ciprofloxacin (4 μg/mL). After 4 h of incubation at 37 °C, both samples and controls were stained with a volume of 1 μL DiBAC(4)3 (0.5 μg/mL) for 30 min, in dark, at 37 °C. Sample reading was performed using the Accuri C6 plus instrument (Becton Dickinson, Biosciences) in the fluorescence channel for FITC. 

Urine samples were excluded from FC analysis if excessive turbidity or blood were noted on visual inspection. Positive/negative control wells containing the standard reference strain (*E. coli* ATCC 25922) grown in the presence/absence of the tested antibiotics were used. Additionally, wells containing only broth/urine diluted in broth, respectively, were included for each urine specimen analyzed.

The urine samples were simultaneously analyzed by the standard method used in the respective clinical unit, consisting in the cultivation of the urine samples on Columbia blood agar and Drigalski Lactose Agar (Becton Dickinson). Both plates were examined for growth after 18–24 h of incubation at 35–37 °C. Urine specimens that presented 10^5^ CFU/mL or more were considered positive, the bacterial isolates being further identified by conventional biochemical methods available in the laboratory and the standard antibiotic susceptibility tests were performed according to CLSI (2018) as described above. The urine samples with no growth or growth less than 10^5^ CFU/mL were considered negative. 

## 3. Results

### 3.1. Antibiotic Susceptibility Profiles of Uropathogenic E. Coli Isolates Cultivated in Sterile Urine Established by Using the Reference Methods versus the FC AST Protocol

The 29 *E. coli* isolates used to optimize the FC AST protocol showed no resistance to nitrofurantoin, 24.13% resistance to trimethoprim–sulfamethoxazole, 41.37% to ceftriaxone, and 55.17% to ciprofloxacin in the disk diffusion assay. The MICs values for each antibiotic were determined using broth microdilution method (CLSI, 2018). The AST was tested using the bacterial suspensions at a final density of 5 × 10^5^ CFU/mL, the capability of the FC protocol to discriminate the threshold of 10^5^ CFU/mL, the most commonly used criteria for defining significant bacteriuria [17,18,19] being previously demonstrated by us [20].

**Figure 1:**
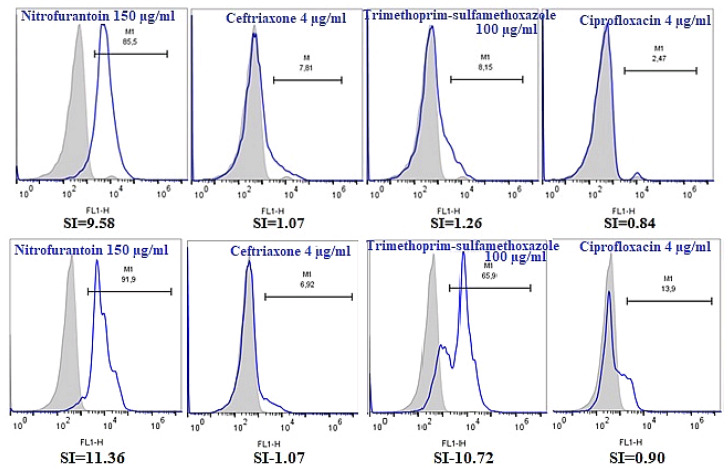
Fluorescence distributions for two *Escherichia coli* isolates grown in artificially contaminated urine treated with antibiotics (nitrofurantoin 150 μg/mL, ciprofloxacin 4 μg/mL, ceftriaxone 4 μg/mL, and trimethoprim–sulfamethoxazole 100 μg/mL). The grey area of the histograms indicates the green fluorescence of untreated bacteria, and the white area with blue contour shows the green fluorescence of bacterial population treated with antibiotics. The fraction of bacterial population with increased green fluorescence in urine sample is expressed in percentages (M1). The SI was defined as the ratio of the median fluorescence of cells treated with antibiotics versus the median fluorescence of untreated cells. The upper histograms correspond to an *E. coli* isolate susceptible to nitrofurantoin (minimum inhibitory concentration (MIC) ≤ 18 μg/mL) and resistant to ceftriaxone (MIC ≥ 4 μg/mL), trimethoprim–sulfamethoxazole (cotrimoxazole) (MIC ≥ 100 μg/mL), and ciprofloxacin (MIC ≥ 4 μg/mL), while the below ones to an *E. coli* isolate susceptible to nitrofurantoin (MIC ≤ 18 μg/mL) and trimethoprim–sulfamethoxazole (MIC ≤ 25 μg/mL) and resistant to ceftriaxone (MIC ≥ 4 μg/mL) and ciprofloxacin (MIC ≥ 4 μg/mL). All these results were in agreement with the standard cultivation method.

Data analysis revealed a good agreement between the MIC determined by broth microdilution and the FC data. For the FC AST assay, the fraction of the bacterial cells with increased green fluorescence due to antibiotic exposure was assessed based upon the green fluorescence distribution of the stained growth control for each *E. coli* isolate. The susceptible *E. coli* isolates exhibited an increased (at least twice) green fluorescence after antibiotic treatment when compared to the untreated control, while in case of the resistant *E. coli* strains, the green fluorescence profile of bacterial cells populations was similar to that of non-exposed bacterial cells, both in artificially contaminated urine samples and broth (Figure 1). 

**Figure 2:**
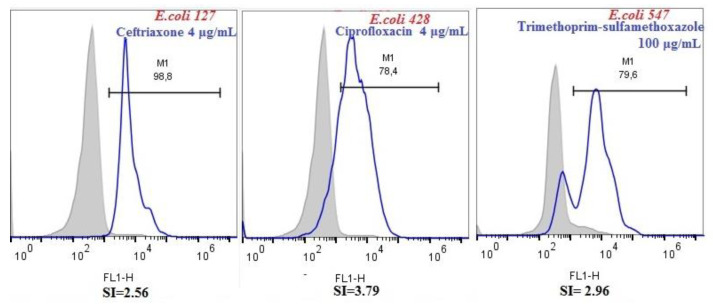
Fluorescence distribution for *E. coli* 127, *E. coli* 428, and *E. coli* 547 isolates. These strains were classified as susceptible to ceftriaxone, ciprofloxacin, and trimethoprim–sulfamethoxazole, respectively, and resistant by standard cultivation method. These results were considered as false-negative.

In a reduced number of cases, the FC AST protocol gave false-negative or false-positive results. For three *E. coli* isolates, i.e., *E. coli* 127, *E. coli* 428, and *E. coli* 547 false-negative results were observed. An enhanced green fluorescence was detected by the instrument indicating depolarization of the membrane, i.e., susceptibility of the tested strains, while being given resistant by the standard cultivation method. The SI values calculated for these *E. coli* isolates were higher than 2 (SI of 2.56 for *E. coli* 127 exposed to 4 μg/mL ceftriaxone, SI of 2.96 for *E. coli* 547 exposed to 100 μg/mL trimethoprim–sulfamethoxazole, and SI of 3.79 for *E. coli* 428 exposed to 4 μg/mL ciprofloxacin) (Figure 2). 

**Figure 3:**
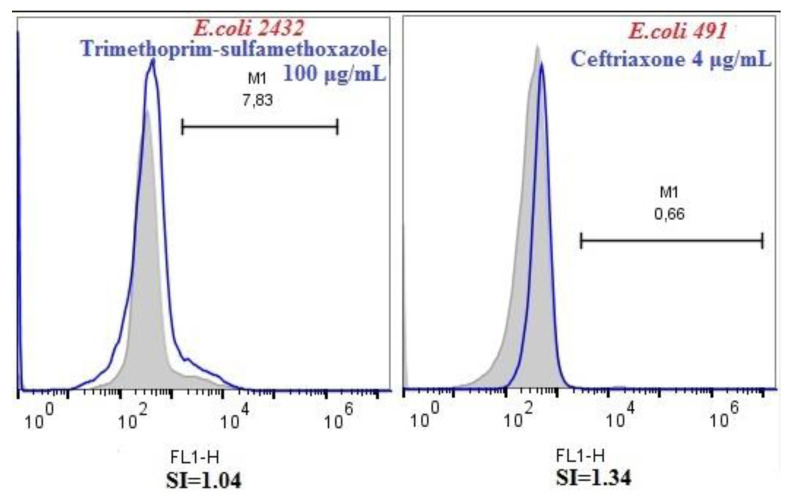
Fluorescence distribution for *E. coli* 2432 and *E. coli* 491 isolates. These strains were classified as resistant to trimethoprim–sulfamethoxazole and, respectively, to ceftriaxone by flow cytometry (FC) and susceptible by cultivation method. These results were considered as false-positive.

In case of two *E. coli* isolates, i.e., *E. coli* 2432 and *E. coli* 491 false-positive results were observed. The fluorescence distributions of the *E. coli* isolates exhibited the same pattern as the isolates not treated with antibiotics. The SI values calculated for these *E. coli* isolates were lower than 2 (SI of 1.04 for *E. coli* 2432 exposed to 100 μg/mL trimethoprim–sulfamethoxazole and SI of 1.34 for *E. coli* 491 exposed to 4 μg/mL ceftriaxone) (Figure 3).

Taken together, the comparative analysis of the AST results obtained for the 29 *E. coli* strains exhibiting different resistance phenotypes using the standard versus FC AST protocols revealed a concordance rate of 100% for nitrofurantoin. Very major discrepancies rates (false-negative or false susceptible isolates) were observed for trimethoprim–sulfamethoxazole (1/7 (14.2%)), ceftriaxone (1/12 (8.3%)), and ciprofloxacin (1/16 (6.25%)). Major discrepancies (false-positive or false resistant isolates), were detected for trimethoprim–sulfamethoxazole (1/22 (4.5%)) and ceftriaxone (1/17 (5.8%)) (Table 1).

### 3.2. Evaluation of Clinical Urine Samples by Using the Optimized FC AST in Comparison with Standard Microbiological Techniques

From the total of 117 collected samples, 10 urine samples were excluded from the FC AST analysis due to the growth of multiple types of colonies on the culture media, indicating urine sample contamination. A total of 77 urine samples (65.8%) were negative (no bacterial population was detected) and 30 were positive (34.18%) (FC protocol was able to detect a viable bacterial population). The results obtained by the FC analysis were 100% in agreement with the standard cultivation method. In case of the 30 microbiologically positive urine sample, standard identification methods revealed the following uropathogens: *E. coli* (16 strains), *Klebsiella pneumoniae* (9 strains); *Proteus mirabilis* (4 strains); *Pseudomonas aeruginosa* (1 strain). 

To bypass the overnight incubation period needed for obtaining single colonies, FC AST was applied directly on the 30 positive UTI specimens infected with the four different bacterial species. With urine samples infected, the FC–AST and standard tests correlated at 100/100 for ceftriaxone and 92.3%/85.7% for ciprofloxacin (Table 2).

Four disagreements were observed with the conventional assays in case of ciprofloxacin, one false-negative result (one infected urine sample with *E. coli*) and three false positive results (one infected urine sample with *K. pneumoniae* and two infected urine samples with *E. coli*).

## 4. Discussion

AST constitutes a prerequisite for the anticipation of microbial resistance phenomenon, a global problem that threatens the effective treatment of infectious diseases [21]. Development of rapid and accurate analysis tools for AST is urgently needed in order to address the high rates of nosocomial morbidity and mortality that occur due to inappropriate, empiric antibiotic treatment [22]. In this context, JPIAMR (Joint Programming Initiative on Antimicrobial Resistance) is addressing this important challenge by launching for 2019 a joint transnational research call on the development of diagnostic and surveillance tools, technologies, and methods to detect antimicrobial resistance (AMR) in clinical and veterinary settings [23]. 

UTIs are among the most common bacterial infections, both in clinical settings and in community [24]. Emergency treatment of UTIs with antibiotics to which the uropathogenic bacteria are already resistant significantly increases the selection and dissemination of resistant pathogens. Additionally, the prescription practices contribute to increasing antibiotic resistance of Gram-negative bacilli to last resort antibiotics, threatening their efficiency in the treatment of severe infections. Therefore, it has become essential that antibiotics should be used judiciously and specifically in the treatment of UTIs [25]. 

Several methods for the rapid detection of bacterial infections aiming to reduce the analysis time have been developed, such MALDI–TOF MS Vitek 2, Vitek MS [9,26,27]. PCR-based techniques, microarrays, microfluidics, whole-genome sequencing were developed to detect antibiotic resistance, but their accuracy remains inferior to that of the conventional phenotypic assays [28]. A rapid AST method was developed that detects an increase in 16S rRNA using a biosensor in 3.5 h analysis time. However, the assay could not be applied for low bacterial loads of the urine sample [29]. Rapid identification and AST profiles of urinary pathogens were also obtained by combining phenotypic (cultivation) and genotypic methods (quantification of DNA) [30]. 

Here, we propose an alternative rapid method based on FC which could provide in 4 h from the arrival of a urine sample in the laboratory, important preliminary information regarding the presence, quantification, and antibiotic resistance profile of uropathogenic *E. coli* strains before the standard analysis results are available. Therefore, using FC for preliminary AST directly in urine specimens could significantly improve the accuracy of emergency antimicrobial therapy in UTIs. We optimized the FC protocol for four antibiotics frequently used in Romania for the treatment of UTIs, i.e., ceftriaxone, ciprofloxacin, nitrofurantoin, and trimethoprim–sulfamethoxazole. Romania is among the top five countries with the highest levels of resistance to third-generation cephalosporins due to ESBL production, the recorded resistance rates being twice as high in Romania (26.8%) than the average value for Europe (13.1%) in 2015 [31]. This trend was ascending, as in 2016, 88.4% of the *E. coli* isolates reported in the annual surveillance report of antimicrobial resistance in Europe were ascertained as ESBL-positive. The spread of ESBL-producing *E. coli* strains is limiting the use of extended-spectrum cephalosporins in UTI infections [32]. Moreover, the increased resistance rates to third-generation cephalosporins are often combined with resistance to fluoroquinolones, which were reported to be of 26–35% in 2016 [33]. In previous studies carried out in Romanian hospital settings, focusing on the characteristics of the fluoroquinolone-resistant *Enterobacteriaceae*, the ESBL phenotype was frequently detected [34,35,36]. The EARSS reports also indicate high resistance rates of bacterial pathogen to beta-lactams, trimethoprim–sulfamethoxazole, and ciprofloxacin, nitrofurantoin being recommended for the treatment of highly resistant pathogens.

Our results confirm previous studies regarding the potential use of FC analysis for prediction of susceptibility of bacteria to antibiotics directly in urine samples, thus significantly reducing the required analysis time [4,6,15,37]. The FC AST assay developed in our study detected antibiotic resistant *E. coli* isolates directly in artificially contaminated urine samples, in 4 h. The proposed FC method uses a fluorescent marker to indicate the alteration of membrane potential. DiBAC4(3), which enters cells with damaged membranes and accumulates in the cytoplasm of depolarized cells, was demonstrated to be a reliable fluorescent viability marker for the assessment of antibiotic susceptibility [15,38,39,40]. In our study, alteration of light scattering signals and depolarization of plasma membrane indicated by an enhanced green fluorescence of DiBAC4(3) were suitable for detection of antibiotic susceptible cells. Based upon the green fluorescence distribution, the developed FC AST protocol could well differentiate, directly in urine samples between antibiotic sensitive and resistant uropathogenic *E. coli* isolates. For each antibiotic, the SI and the percent of depolarized population were determined. The assay results indicated that the number of DiBAC4(3) stained cells increased significantly in populations of sensitive *E. coli* strains treated with the tested antibiotics after 4 h incubation time, whereas the cells of resistant strains were not stained by the dye; consequently the percentage of the depolarized cells remained very low, similar to that of the untreated control. Comparing the results of proposed protocol for AST based upon FC AST assay with those obtained by the reference method, the interpretive category results matched for 29 out of 29 isolates in case of nitrofurantoin, 27 out of 29 for ceftriaxone, 27 out of 29 for trimethoprim–sulfamethoxazole, and 28 out of 29 for ciprofloxacin. 

False-positive or false-negative results were therefore observed in a limited number of cases. The validation of the FC AST method on clinical urine samples collected from in-patients included two broad-spectrum antibiotic classes, frequently recommended for the emergency treatment of UTIs, i.e., fluoroquinolones (ciprofloxacin) and cephalosporins (ceftriaxone) [41,42,43]. The comparative analysis of the FC results with the reference method indicated 100% agreement between the results obtained by the standard assays and those provided by FC AST for ceftriaxone and only a few discrepancies for ciprofloxacin. It is known that drugs commonly act to increase the membrane permeability, reducing the proton-motive force. As a consequence, bacteria developed a resistance mechanism consisting in the reduction of the net negative charge of the bacterial outer membrane, which consequently decreases the binding affinity of antibiotics to the bacterial surface [44]. However, studies have indicated that for very similar values of the electrical potential difference across the membrane of *E. coli*, variations of 10-fold difference in MIC were recorded suggesting that other factors may also play a role in drug susceptibility [45]. Moreover, the altered membrane depolarization may not always lead to death. Depending on the degree of alteration, the functioning of the cell may be affected [46,47]. Some other studies reported that membrane potential alteration could be reversible, bacteria being able to recover when placed in fresh medium [48]. 

## 5. Conclusions

We propose here an alternative method based on FC, which can provide important preliminary information regarding the bacterial presence in urine and the antibiotic resistance profile of uropathogenic *E. coli* strains. The FC based AST protocol was optimized for detecting uropathogenic *E. coli* in sterile urine samples artificially contaminated with a final density of 5 × 10^5^ CFU/mL and to differentiate among their susceptibility or resistance to ciprofloxacin, trimethoprim–sulfamethoxazole, nitrofurantoin, and ceftriaxone after 4 h of incubation versus minimum 24 h required by the conventional AST assays. The FC based AST protocol was validated on clinical urine samples collected from inpatients for two broad spectrum antibiotics frequently recommended for the emergency therapy of UTIs, i.e., ciprofloxacin and ceftriaxone. The early information regarding the antibiotic susceptibility profiles provided by the direct analysis of clinical specimens could potentially contribute to a more targeted empiric therapy in the anticipation of reference AST results. Thus, the FC AST could prevent the emergence and dissemination of drug resistant pathogens and reduce the morbidity and mortality rates associated with inappropriate emergency therapy in UTIs.

## Figures and Tables

**Table 1:** Comparative results of the antibiotic susceptibility testing (AST) profiles of the analyzed *E. coli* strains established using the FC AST protocol and the standard broth dilution method.

Strains	Nitrofurantoin		Trimethoprim-SulfamethoxaZole		Ceftriaxone		Ciprofloxacin	
	FC AST	Standard AST	FC AST	Standard AST	FC AST	Standard AST	FC AST	Standard AST
*E. coli 428*	S	S	S	S	S	S	S	R
*E. coli127*	S	S	S	S	S	R	R	R
*E. coli 956*	S	S	R	R	R	R	R	R
*E. coli 4493*	S	S	S	S	R	R	R	R
*E. coli 451*	S	S	S	S	R	R	R	R
*E. coli 424*	S	S	S	S	R	R	R	R
*E. coli 547*	S	S	S	R	S	S	S	S
*E. coli 491*	S	S	R	R	R	S	S	S
*E. coli 3894*	S	S	S	S	S	S	S	S
*E. coli 3830*	S	S	R	R	R	R	R	R
*E. coli 213*	S	S	R	R	S	S	R	R
*E. coli 3812*	S	S	R	R	S	S	R	R
*E. coli 220*	S	S	S	S	S	S	R	R
*E. coli 253*	S	S	S	S	S	S	R	R
*E. coli 214*	S	S	S	S	S	S	S	S
*E. coli 8426*	S	S	S	S	S	S	S	S
*E. coli 130*	S	S	S	S	S	S	S	S
*E. coli 429*	S	S	S	S	S	S	S	S
*E. coli 3865*	S	S	S	S	R	R	R	R
*E. coli 27*	S	S	S	S	R	R	R	R
*E. coli 102*	S	S	R	R	R	R	R	R
*E. coli 2448*	S	S	S	S	R	R	S	S
*E. coli 3906*	S	S	S	S	S	S	S	S
*E. coli 2498*	S	S	S	S	R	R	R	R
*E. coli 2416*	S	S	S	S	S	S	S	S
*E. coli 218*	S	S	S	S	R	R	R	R
*E. coli 2432*	S	S	R	S	S	S	S	S
*E. coli 439*	S	S	S	S	S	S	S	S
*E. coli 2415*	S	S	S	S	S	S	S	S
Sensitivity (%) a/(a+c)	100		85.7		91.6		93.7	
Specificity (%) d/(b+d)	100		95.6		94.1		100	
No. of major discrepancy/no. of susceptible isolates (%)	0		1/22 (4.5)		1/17 (5.8)		0	
No. of very major discrepancy/no. of resistant isolates (%)	0		1/7 (14.2)		1/12 (8.3)		1/16 (6.25)	

Legend: a = true positive (resistant), b = false-positive, c = false-negative, d = true negative (sensitive). S = sensitive, R = resistant. The MICs values for nitrofurantoin were: S < 18 μg/mL, R >150 μg/mL; for trimethoprim–sulfamethoxazole: S < 25 μg/mL, R > 100 μg/mL; for ceftriaxone S < 1 μg/mL, R ≥ 4 μg/mL; and for ciprofloxacin S < 1 μg/mL, R ≥ 4 μg/mL.

**Table 2:** Comparative results of the AST results of the analyzed infected urinary tract infection (UTI) specimens using the FC AST protocol and the standard broth dilution method.

Urine Samples	No of Urine Samples Tested	Ceftriaxone				Ciprofloxacin			
		No of Urine Samples Tested Resistant		No of Urine Samples Tested Susceptible		No of Urine Samples Tested Resistant		No of Urine Samples Tested Susceptible	
		FC AST	Standard AST	FC AST	Standard AST	FC AST	Standard AST	FC AST	Standard AST
*E. coli*	16	2	2	14	14	7	6	9	10
*K. pneumoniae*	9	2	2	7	7	4	3	5	6
*P. mirabilis*	4	3	3	1	1	3	3	1	1
*P. aeruginoasa*	1	1	1	0	0	1	1	0	0
Sensitivity (%)		100				92.3			
Specificity (%)		100				85.7			
No. of major discrepancy/no. of susceptible isolates (%)		0				14.2			
No. of very major discrepancy/no. of resistant isolates (%)		0				7.6			

## References

[1] Davenport M., Mach K.E., Shortliffe L.M.D., Banaei N., Wang T.-H., Liao J. (2017). New and developing diagnostic technologies for urinary tract infections. Nat. Rev. Urol..

[2] Faria-Ramos I., Espinar M., Rocha R., Santos-Antunes J., Rodrigues A.G., Canton R., Pina-Vaz C. (2013). A novel flow cytometric assay for rapid detection of extended-spectrum beta-lactamases. Clin. Microbiol. Infect..

[3] Silva A.P., Faria-Ramos I., Ricardo E., Miranda I.M., Espinar M.J., Costa-De-Oliveira S., Canton R., Rodrigues A.G., Pina-Vaz C. (2016). Rapid Flow Cytometry Test for Identification of Different Carbapenemases in Enterobacteriaceae. Antimicrob. Agents Chemother..

[4] Costa-De-Oliveira S., Teixeira-Santos R., Silva A.P., Pinho E., Mergulhão P., Silva-Dias A., Marques N., Martins-Oliveira I., Rodrigues A.G., Paiva J.A. (2017). Potential Impact of Flow Cytometry Antimicrobial Susceptibility Testing on the Clinical Management of Gram-Negative Bacteremia Using the FASTinov® Kit. Front. Microbiol..

[5] Van der Zwet W.C., Hessels J., Canbolat F., Deckers M.M. (2010). Evaluation of the Sysmex UF-1000i® urine flow cytometer in the diagnostic work-up of suspected urinary tract infection in a Dutch general hospital. Clin. Chem. Lab. Med..

[6] Broeren M.A.C., Bahçeci S., Vader H.L., Arents N.L.A. (2011). Screening for Urinary Tract Infection with the Sysmex UF-1000i Urine Flow Cytometer. J. Clin. Microbiol..

[7] Geerts N., Jansz A., Boonen K., Wijn R., Koldewijn E.L., Boer A.-K., Scharnhorst V. (2015). Urine flow cytometry can rule out urinary tract infection, but cannot identify bacterial morphologies correctly. Clin. Chim. Acta.

[8] Monsen T.J., Rydén P. (2014). Flow Cytometry Analysis Using Sysmex UF-1000i Classifies Uropathogens Based on Bacterial, Leukocyte, and Erythrocyte Counts in Urine Specimens among Patients with Urinary Tract Infections. J. Clin. Microbiol..

[9] Zboromyrska Y., Rubio E., Alejo I., Vergara A., Mons A., Campo I., Bosch J., Marco F., Vila J. (2016). Development of a new protocol for rapid bacterial identification and susceptibility testing directly from urine samples. Clin. Microbiol. Infect..

[10] Monsen T.J., Ryden P. (2017). A new concept and a comprehensive evaluation of SYSMEX UF-1000i flow cytometer to identify culture-negative urine specimens in patients with UTI. Eur. J. Clin. Microbiol. Infect. Dis..

[11] Kamerzan C., Ciubucă B., Dincă G., Bleotu C., Drumea V., Chifiriuc M., Popa M., Pîrcălăbioru G.G., Marutescu L., Lazar V. (2017). Development and Sequential Analysis of a New Multi-Agent, Anti-Acne Formulation Based on Plant-Derived Antimicrobial and Anti-Inflammatory Compounds. Int. J. Mol. Sci..

[12] Marutescu L., Calu L., Chifiriuc M., Bleotu C., Daniliuc C.G., Fălcescu D., Kamerzan C., Badea M., Olar R. (2017). Synthesis, Physico-chemical Characterization, Crystal Structure and Influence on Microbial and Tumor Cells of Some Co(II) Complexes with 5,7-Dimethyl-1,2,4-triazolo[1,5-a]pyrimidine. Molecules.

[13] Scăețeanu G.V., Chifiriuc M., Bleotu C., Kamerzan C., Marutescu L., Daniliuc C.G., Maxim C., Calu L., Olar R., Badea M. (2018). Synthesis, Structural Characterization, Antimicrobial Activity, and In Vitro Biocompatibility of New Unsaturated Carboxylate Complexes with 2,2′-Bipyridine. Molecules.

[14] Clinical and Laboratory Standards Institute (2018). Performance Standards for Antimicrobial Susceptibility Testing.

[15] Nuding S., Zabel L.T. (2013). Detection, Identification, and Susceptibility Testing of Bacteria by Flow Cytometry. J. Bacteriol. Parasitol..

[16] Garcia L.S., Isenberg H.D. (2010). Clinical Microbiology Procedures Handbook.

[17] Kass E.H. (1957). Bacteriuria and the Diagnosis of Infections of the Urinary Tract. AMA. Arch. Intern. Med..

[18] Stamm W.E., Counts G.W., Running K.R., Fihn S., Turck M., Holmes K.K. (1982). Diagnosis of Coliform Infection in Acutely Dysuric Women. N. Engl. J. Med..

[19] Wilson M.L., Gaido L. (2004). Laboratory Diagnosis of Urinary Tract Infections in Adult Patients. Clin. Infect. Dis..

[20] Velican A.M., Kamerzan C., Maruțescu L., Lambert C., Chifiriuc M.C. (2019). The development of an analysis protocol based on flow cytometry for rapid detection of uropathogenic E. coli in artificially contaminated urine samples. Rom. Biotechnol. Lett..

[21] Premanandh J., Samara B.S., Mazen A.N. (2016). Race against Antimicrobial Resistance Requires Coordinated Action–An Overview. Front. Microbiol..

[22] Lee C.-R., Cho I.H., Jeong B.C., Lee S.H. (2013). Strategies to Minimize Antibiotic Resistance. Int. J. Environ. Res. Public Health.

[23] https://www.jpiamr.eu/now-open-call-on-amr-diagnostics-and-surveillance/.

[24] Flores-Mireles A.L., Walker J.N., Caparon M., Hultgren S.J. (2015). Urinary tract infections: Epidemiology, mechanisms of infection and treatment options. Nat. Rev. Genet..

[25] Schmiemann G., Kniehl E., Gebhardt K., Matejczyk M.M., Hummers-Pradier E. (2010). The Diagnosis of Urinary Tract Infection. Dtsch. Aerzteblatt Online.

[26] Ferreira L., Vega Castaño S., Sánchez-Juanes F., González M., Herrero A., Muñiz M.C., González-Buitrago J.M., Muñoz J.L. (2010). Identifying bacteria using a matrix-assisted laser desorption ionization time-of-flight (MALDI-TOF) mass spectrometer. Comparison with routine methods used in clinical microbiology laboratories. Enferm. Infecc. Microbiol. Clin..

[27] Huang B., Zhang L., Zhang W., Liao K., Zhang S., Zhang Z., Ma X., Chen J., Zhang X., Qu P. (2017). Direct Detection and Identification of Bacterial Pathogens from Urine with Optimized Specimen Processing and Enhanced Testing Algorithm. J. Clin. Microbiol..

[28] Pulido M.R., García-Quintanilla M., Martín-Peña R., Cisneros J.M., McConnell M.J. (2013). Progress on the development of rapid methods for antimicrobial susceptibility testing. J. Antimicrob. Chemother..

[29] Mach K.E., Mohan R., Baron E.J., Shih M.-C., Gau V., Wong P.K., Liao J. (2011). A Biosensor Platform for Rapid Antimicrobial Susceptibility Testing Directly From Clinical Samples. J. Urol..

[30] Mezger A., Gullberg E., Göransson J., Zorzet A., Herthnek D., Tano E., Nilsson M., Andersson D.I. (2014). A General Method for Rapid Determination of Antibiotic Susceptibility and Species in Bacterial Infections. J. Clin. Microbiol..

[31] European Public Health Alliance (2017). In the Red Zone–Antimicrobial Resistance: Lessons from Romania Brussels.

[32] Ciontea A.S., Cristea D., Andrei M.M., Popa A., Usein C.R. (2018). In vitro antimicrobial resistance of urinary Escherichia coli isolates from outpatients collected in a laboratory during two years, 2015–2017. Roum. Arch. Microbiol. Immunol..

[33] Gouvras G. (2004). The European Centre for Disease Prevention and Control. Eurosurveillance.

[34] Mitache M.M., Curutiu C., Rusu E., Bahna R., Ditu M., Moldovan H., Hancu V., Chifiriuc M.C. (2017). Urinary Tract Infections in Patients with Type 1 and Type 2 Diabetes: Etiology, Resistance to Antibacterial Chemicals and Virulence Features. Rev. Chim..

[35] Trușcă B.-S., Gheorghe I., Marutescu L., Curutiu C., Marinescu F., Ghiță C.M., Borcan E., Țuică L., Minciuna V., Gherghin H.-E. (2017). Beta-lactam and quinolone resistance markers in uropathogenic strains isolated from renal transplant recipients. Rev. Romana Med. Lab..

[36] Usein C.-R., Tatu-Chiţoiu D., Nica M., Ciontea S.A., Palade A.-M., Condei M., Damian M. (2009). Characteristics of Romanian fluoroquinolone-resistant human clinical Escherichia coli isolates. Roum. Arch. Microbiol. Immunol..

[37] Gauthier C., St-Pierre Y., Villemur R. (2002). Rapid antimicrobial susceptibility testing of urinary tract isolates and samples by flow cytometry. J. Med. Microbiol..

[38] Lebaron P., Joux F. (2000). Review Use of fluorescent probes to assess physiological functions of bacteria at single-cell level. Microbes Infect..

[39] Léonard L., Chibane L.B., Bouhedda B.O., Degraeve P., Oulahal N. (2016). Recent Advances on Multi-Parameter Flow Cytometry to Characterize Antimicrobial Treatments. Front. Microbiol..

[40] Saint-Ruf C., Crussard S., Franceschi C., Orenga S., Ouattara J., Ramjeet M., Surre J., Matic I. (2016). Antibiotic Susceptibility Testing of the Gram-Negative Bacteria Based on Flow Cytometry. Front. Microbiol..

[41] Alanazi M.Q. (2018). An evaluation of community-acquired urinary tract infection and appropriateness of treatment in an emergency department in Saudi Arabia. Ther. Clin. Risk Manag..

[42] Bischoff S., Walter T., Gerigk M., Boutros M., Vogelmann R. (2018). Empiric antibiotic therapy in urinary tract infection in patients with risk factors for antibiotic resistance in a German emergency department. BMC Infect. Dis..

[43] CDC (2019). Antibiotic Use in the United States, 2018 Update: Progress and Opportunities.

[44] Andersson D.I., Hughes D., Kubicek-Sutherland J., Kubicek-Sutherland J.Z. (2016). Mechanisms and consequences of bacterial resistance to antimicrobial peptides. Drug Resist. Updat..

[45] Damper P.D., Epstein W. (1981). Role of the membrane potential in bacterial resistance to aminoglycoside antibiotics. Antimicrob. Agents Chemother..

[46] Halder S., Yadav K.K., Sarkar R., Mukherjee S., Saha P., Haldar S., Karmakar S., Sen T. (2015). Alteration of Zeta potential and membrane permeability in bacteria: A study with cationic agents. SpringerPlus.

[47] Esposito F., Fernandes M., Lopes R., Muñoz M., Sabino C.P., Cunha M., Silva K.C., Cayô R., Martins W.M., Moreno A.M. (2017). Detection of Colistin-Resistant MCR-1-Positive Escherichia coli by Use of Assays Based on Inhibition by EDTA and Zeta Potential. J. Clin. Microbiol..

[48] Nebe-Von-Caron G., Stephens P.J., Hewitt C.J., Powell J.R., Badley R.A. (2000). Analysis of bacterial function by multi-colour fluorescence flow cytometry and single cell sorting. J. Microbiol. Methods.

